# Application of fecal microbial transplantation in hepatic encephalopathy after transjugular intrahepatic portosystemic shunt

**DOI:** 10.1097/MD.0000000000028584

**Published:** 2022-01-21

**Authors:** Jun Li, Dan Wang, Jingping Sun

**Affiliations:** Department of Gastroenterology, Clinical Medical College and the First Affiliated Hospital of Chengdu Medical College, Chengdu, China.

**Keywords:** fecal microbiota transplantation, hepatic encephalopathy, intestinal flora, transjugular intrahepatic portosystemic shunt

## Abstract

**Rationale:**

Transjugular intrahepatic portosystemic shunt (TIPS) is mainly used to treat acute and chronic esophageal, gastric, and intestinal variceal bleeding and refractory ascites caused by portal hypertension. The most common complication of TIPS is the development of hepatic encephalopathy (HE). Fecal microbiota transplantation (FMT) is an emerging method for treating diseases by altering the intestinal flora. We present 2 cases of FMT that ameliorated liver function and HE after TIPS.

**Patient concerns:**

In this report, 2 patients with liver cirrhosis secondary to hepatitis B had recurrent Grade 2-3 HE after TIPS.

**Diagnosis:**

Two patients were diagnosed as having HE.

**Interventions:**

The 2 patients separately received 3 times of FMT.

**Outcomes:**

The liver function of both patients improved, the clinical symptoms were relieved, and the number of HE attacks decreased significantly after FMT.

**Lessons:**

FMT may be another effective way to treat HE, and is worthy of further research.

## 1 Introduction

In the decompensated stage of liver cirrhosis, patients experience a continuous decline in liver function and an increase in portal vein pressure, which can easily induce a variety of complications. Variceal bleeding caused by portal hypertension is a serious and life-threatening complication in patients with liver cirrhosis.^[1]^ Transjugular intrahepatic portosystemic shunt (TIPS) is an established treatment for the complications of portal hypertension. Currently, the postoperative complications of TIPS include hepatic encephalopathy (HE), abdominal hemorrhage, nonsurgical infection, and heart failure. And 29% to 60% of patients develop HE within 1 year. Unfortunately, to date, no drug treatment has been shown to reduce the incidence of HE after TIPS. The high incidence of HE after TIPS and poor therapeutic effects have become the main limiting factors in the clinical application of TIPS.

HE is a serious neuropsychiatric disorder associated with acute or chronic liver disease, the exact pathogenesis of which has not yet been determined. Certain mechanisms have been recognized for years, including increased production of neurotoxins, impairment of neurotransmission, systemic inflammation, alteration of the blood–brain barrier, and alterations in energy metabolism.^[2]^ The Ammonia generated by the intestinal flora is a critical driver of HE. Therefore, balancing flora disorders is a potential therapeutic strategy. Currently, some diseases related to intestinal flora disorders can be treated through fecal microbiota transplantation (FMT). FMT involves the transplantation of intestinal flora in the feces of healthy people into the gastrointestinal tract of patients so that patients can rebuild the new intestinal flora to achieve the treatment of intestinal and extra-intestinal diseases. FMT has been proven to be a safe and effective treatment for *Clostridium difficile* infection (CDI) and is recommended for recurrent or refractory infections.^[3]^ Besides, as reported previously, 1 studies have shown that FMT can be used to treat or manage HE in patients with cirrhosis.^[1]^ However, studies on FMT to treat HE after TIPS are rare. As dysbiosis is thought to contribute to HE, we hypothesized that FMT could ameliorate HE after TIPS. This study was registered at ClinicalTrials. gov (ID: NCT03013712). This study was performed in accordance with the nation's relevant laws and approved by the ethics committee of the First Affiliated Hospital of Chengdu Medical College (2017009).

Based on the 2 cases of FMT, this report further explores the mechanism, existing problems, and prospects of FMT.

## 2 Case presentation

**Figure 1:**
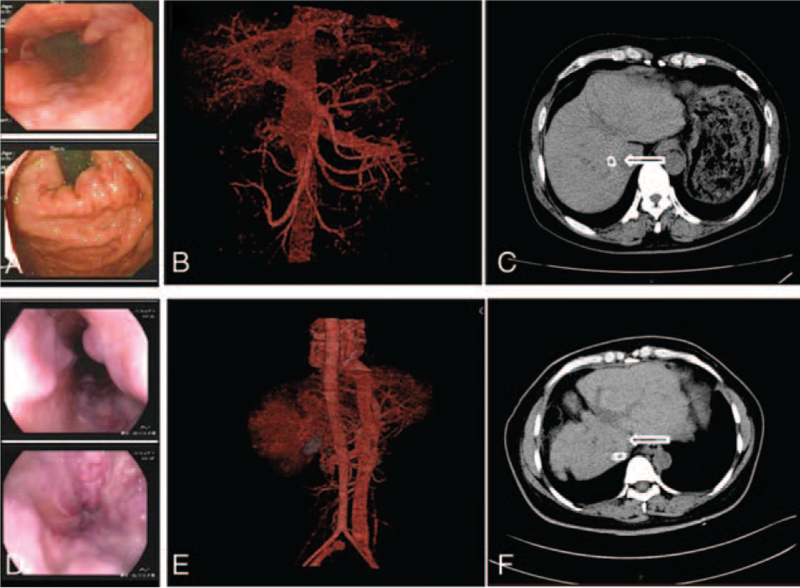
Laboratory examinations of case 1 and case 2. (A,D) preoperative case 1 and case 2 endoscopic esophagus and gastric fundus, (B,E) preoperative abdominal angiography, (C,F) abdominal CT after TIPS. CT = computed tomography, TIPS = transjugular intrahepatic portosystemic shunt.

**Case 1**: A 58-year-old male patient with a history of hepatitis B for 23 years, liver cirrhosis and recurrent ascites for 10 years, and lamivudine as antiviral therapy for 9 years, after which lamivudine was replaced with adefovir dipivoxil. Gastroscopy revealed 4 varicose veins in the middle and lower esophagus were about 0.6 cm, esophageal varicose veins (severe, RC+), gastric varicose veins, and portal hypertension gastropathy (Fig. 1A). Abdominal angiography showed a slightly widened portal vein, varicose veins around the gastric fundus and spleen, and a flaky abnormal perfusion shadow in the right lobe of the liver (Fig. 1B). As a result of repeated hematemesis and menela, he accepted TIPS treatment in December 16, 2016. The stent implantation was successful (Fig. 1C). The patient was provided strict dietary management and was administered lactulose, aspartate, and ornithine orally after TIPS to prevent the occurrence of HE. However, he suffered from Grade 2 to 3 HE 3 times in the first month after the operation. After obtaining the patient's written informed consent and completing routine examination to ensure that there were no contraindications to FMT, we performed FMT under gastroscopy 3 times in total to prevent HE recurrence. Approximately 50 g of fresh fecal intestinal flora suspension was implanted once. Random serum ammonia, hepatic enzyme, and bilirubin levels were measured during each visit. The patient was advised to maintain a similar diet and lactulose dose throughout the study.

**Case 2:** A 43-year-old woman with a history of hepatitis B for 18 years, recurrent ascites for 18 years, liver cirrhosis for 4 years, and adefovir dipivoxil as antiviral therapy for 14 years. Gastroscopy revealed 4 varicose veins in the lower esophagus were about 0.5 cm, esophageal variceal veins, gastric varicose veins, and portal hypertension gastropathy (Fig. 1D). Abdominal angiography showed that the main portal vein and the superior mesenteric vein widened, and multiple varicose veins in the esophagus, stomach, and spleen; a flaky abnormal perfusion shadow was observed in the right lobe of the liver (Fig. 1E). Because of refractory ascites and repeated hematochezia, she underwent TIPS treatment in December 13, 2016. The stent implantation was successful (Fig. 1F). Strict dietary management and routine administration of lactofructose and aspartate ornithine were administered to prevent HE. In the first month, she suffered from grade 2 to 3 HE 2 times. To prevent recurrent HE, the same FMT as that in Case 1 was performed. Random serum ammonia, hepatic enzyme, and bilirubin levels were measured during each visit. The patient was advised to maintain a similar diet and lactulose dose throughout the study.

## 3 Outcome and follow-up of patients

After discharge, patients were advised to continue half-dose lactulose therapy. During the follow-up period of nearly 1 year, the patients were not hospitalized due to HE again, and there were no clinical manifestations such as hematemesis, menela, and ascites, and the blood ammonia level decreased significantly. The liver function in Case 1 was better than before. Although the liver function of the latter group was not significantly improved, no obvious clinical symptoms were observed. Child–Pugh scores decreased significantly after the 3 FMTs (Table 1). There were no FMT-related adverse events or infection complications in case 1. Temporary constipation occurred for 7 days in case 2 after FMT, and no other FMT-related adverse events or infection complications occurred. They were satisfied with the results of the study.

**Table 1:** The levels of serum ammonia, liver enzyme and bilirubin were measured randomly at each visit.

	Case 1				Case 2			
	Basic	FMT1	FMT2	FMT3	Basic	FMT1	FMT2	FMT3
Date	February 20, 2019	March 6, 2019	April 1, 2019	May 6, 2019	February 23, 2019	March 7, 2019	April 7, 2019	May 10, 2019
HE (/mo)	3 times	1 time	No	No	2 times	1 time	No	No
NH3 (μmol/L)	104.2	73.9	91.1	65.8	107.3	99.8	80.1	71.1
Ascites	Middle	Little	Little	No	Little	Little	No	No
AST (U/L)	11	13	12	15	22	67	44	95
ALT (U/L)	41	50	47	35	36	52	60	44
PT (S)	14.7	13.5	14.5	13.2	16.6	15.5	16.0	14.3
T-BIL (μmol/L)	33.2	57.7	34.3	31.7	55.6	56.4	36.5	40.7
ALB (g/L)	30.6	33.4	32.5	35.6	33.3	32.4	28.5	31.7
Child–Pugh score	10	10	8	5	11	10	7	7

## 4 Results of 16s RNA analysis of donors and patients

**Figure 2:**
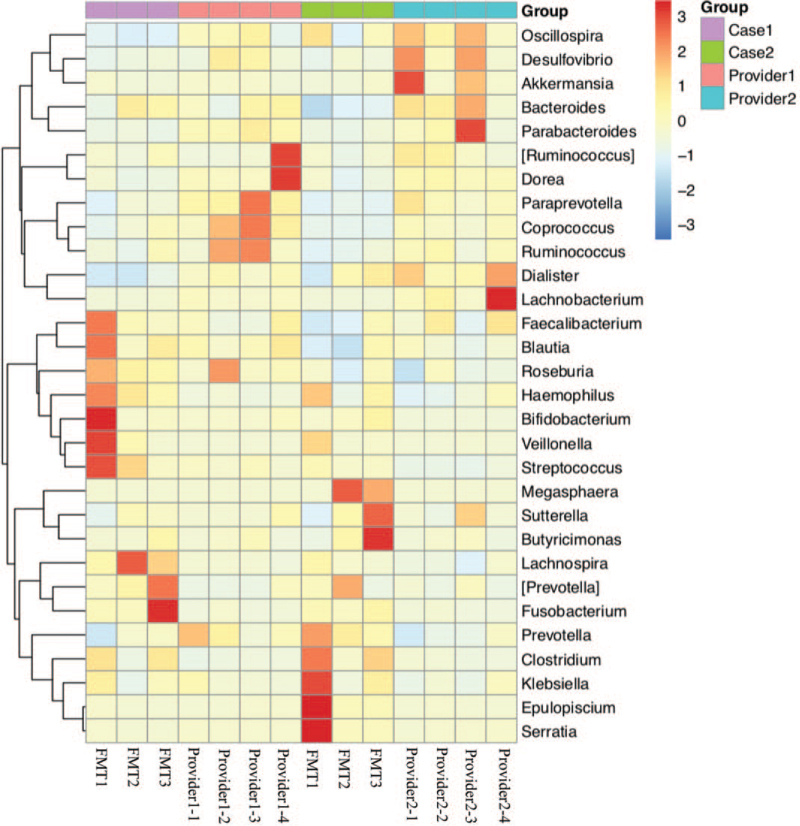
The top 30 species were selected from the specimens and heat maps were drawn for display. FMT = fecal microbiota transplantation.

**Figure 3:**
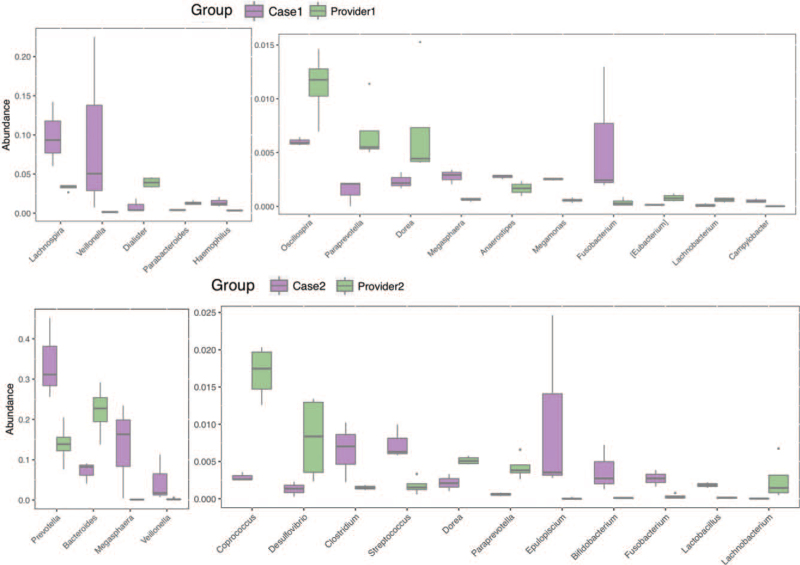
The abundance of the intestinal microbiota in donors and patients. In terms of genus abundance table, rank-sum test +FDR correction was used to identify 21 differential species in the case 1 and provider 1 groups, and 24 differential species in the case 2 and provider 2 groups. The top 15 differential species were identified.

**Figure 4:**
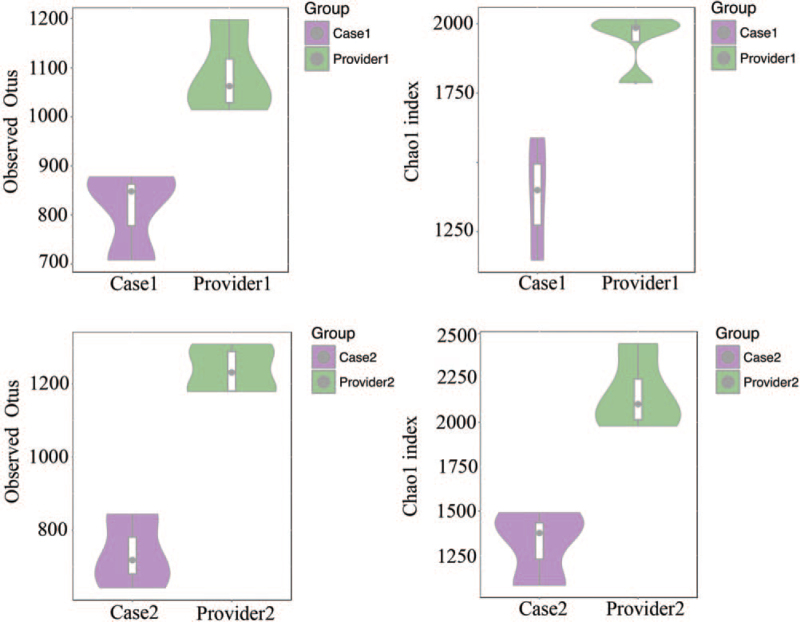
The diversity of the intestinal microbiota in donors and patients.

**Figure 5:**
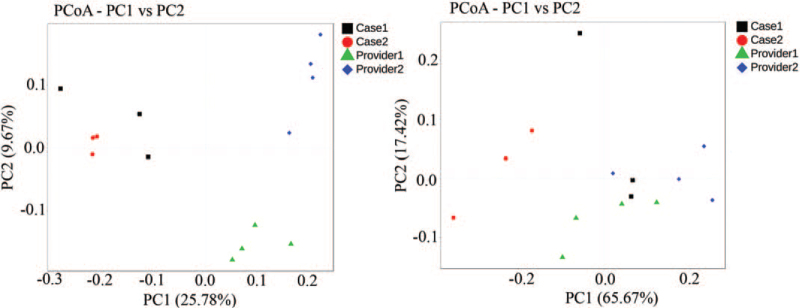
Differences in overall species composition. On the left is the PCoA analysis result based on unweighted unifrac distance; on the right is the PCoA analysis result based on weighted unifrac distance. PcoA = Principal co-ordinates analysis, PC = Principal component scores.

Stool samples were obtained from the patient before FMT (basic) and 1 week after each FMT. Microbiota DNA was extracted using a genomic DNA extraction kit (TIANGEN). Specific primers 341F (5′-CCTACACGACGCTCTTCCGATCTN-3′) and 805R (5′-GACTGGAGTTCCTTGGCACCCGAGAATTCCA-3′) were used to perform PCR amplification of the 16s V3-V4 region. After comparison, 16S sequencing was performed on the Illumina Hiseq-PE250 technology sequencing platform to perform 16S V3-V4 region sequencing of the sample by the double-end sequencing method, and the corresponding data were statistically analyzed according to the sequencing results. The heat map is drawn based on the abundance of fecal microbiota composition of donors and patient samples at the genus level (Fig. 2). Beneficial bacteria, such as *Ruminococcus, Akkermansia*, and *Oscillospir*, were highly abundant in the donors, which had a low abundance in the patients before FMT. Some pathogenic and opportunistic bacteria, such as *Veillonella* and *Megasphaera*, were less abundant in the donors. In terms of the genus abundance table, the abundance of donor intestinal flora was higher than that of patients, and the donor and patient communities were clustered into 1 group (Fig. 3). We used 2 indices of community abundance (Chao1 and observed Otus) to measure the diversity of intestinal microbiota in donors and patients. Overall, we observed an increase in the diversity of both indices from patients to donors, and this trend in microbiome diversity suggests that donors’ intestinal flora is more diverse than that of patients (Fig. 4). PCoA analysis found that there are significant differences in overall species composition (Fig. 5). The composition of the intestinal flora changed after FMT, indicating that FMT effectively regulated the flora structure in patients.

## 5 Discussion

To the best of our knowledge, there are few reports on the use of FMT for the treatment of HE. In this study, we used FMT to treat recurrent HE after TIPS. In this case, 2 Child–Pugh class C patients with post-HBV cirrhosis were treated with FMT through gastroscopy because of severe varicose veins and frequent HE after TIPS. The intestinal flora changes in the patients after transplantation are obvious, their condition has also improved, their incidence rate of hepatic encephalopathy has decreased, and Child–Pugh scores have fallen. However, patients were followed up for just 1 year; the follow-up effect is uncertain for the time being, and continuous follow-up is needed.

HE is a serious neuropsychiatric disorder of acute or chronic liver disease, and the focus of HE treatment is to reduce ammonia production, promote ammonia clearance, and reduce inflammatory reactions. Lactulose is a commonly used drug for this purpose. Its therapeutic mechanism is to acidify the intestine, promote the growth of probiotics (such as *Lactobacillus* and *Bifidobacteria*), and inhibit glutamine and ammonia metabolism.^[4]^ Antibiotics such as rifaximin, a broad-spectrum antibiotic, can inhibit the growth of gram-positive, gram-negative, and intestinal anaerobic bacteria. Its mechanism of action is to combine with DNA-dependent RNA polymerase, block RNA synthesis, and inhibit bacterial growth.^[5]^ As reported previously, 4 L-ornithine-L-aspartate (LOLA) activates glutamine synthetase activity in skeletal muscle, promotes glutamine synthesis, and increases ammonia clearance.

In recent years, it has been increasingly recognized that intestinal flora plays a significant role in the development of diseases. Thus, changing intestinal flora through FMT may become another treatment method for HE. The adverse effects of intestinal flora disorders on ammonia metabolism and inflammation can be eliminated by ft-implantation of healthy intestinal flora. Bajaj et al^[6]^ showed a significant difference in the mucosal microbiome between HE and non-HE patients. Specifically, *Firmicutes*, such as members of genera *Veillonella*, *Megasphaera*, *Bifidobacterium*, and *Enterococcus*, were higher in HE, whereas *Roseburia was* more abundant in the no-HE group. *Blautia*, *Fecalibacterium*, *Roseburia*, and *Dorea* were all associated with good cognitive ability and reduced inflammation, whereas *Enterococcus*, *Megasphaera*, and *Burkholderia*, which were overexpressed in HE, were associated with poor cognition and aggravated inflammation. A patient with hepatitis C developed HE who received FMT once a week for a total of 4 times, and his cognitive ability was significantly improved, and the microbiota composition was also clustered with the donor.^[7]^ Bajaj et al^[8]^ performed FMT in 20 patients with HE complicated with cirrhosis, who were not hospitalized for HE compared with the control group, and no adverse events occurred after FMT; the relative abundance of the bacteria increased, while *Ruminococcus* and *Lachnospiraceae* significantly increased and *Firmicutes* were relatively expanded. These results indicate that FMT is a new direction for the treatment of HE. Other studies have shown that sterile suspensions of donor feces are sufficient to restore normal fecal habits and eliminate symptoms only in those infected with *Clostridium difficile*.^[9]^ The substance in the aseptic suspension is still generated by bacteria, and only a sterile fecal suspension is implanted. After the matter in the suspension was metabolized, this effect gradually disappeared. Therefore, FMT is a long-term treatment method.

Most previous studies have used colonoscopy, enema, and gastroscopy. In this case, the FMT was performed using a gastroscope. Colonoscopy and enema are designed to implant fecal suspensions into the large intestine, whereas only gastroscopy can place fecal suspensions in the ileocecal part to the maximum extent. The ileocecal part is the junction of the small intestine and the large intestine, which makes it easy for bacteria to grow up to the small intestine, leading to excessive growth of the intestinal flora. FMT frequency includes once a week, once every 2 weeks, once a month, and only once. Currently, there is no study on the influence of FMT frequency on the FMT effect.

Therefore, FMT is a promising emerging therapy. FMT could restore the diversity of intestinal flora in patients to achieve a therapeutic effect, and it has the advantages of low cost and easy popularization. At present, FMT is widely used in severe and complex *Clostridium difficile* infections. Its application in clinical treatment has proven to be safe and effective. However, its application is far from being developed and requires further exploration.

## Author contributions

**Conceptualization**: Dan Wang, Jun Li, Jingping Sun.

**Data curation**: Dan Wang.

**Formal analysis**: Jun Li.

**Funding acquisitio**n: Jingping Sun.

**Investigation**: Dan Wang, Jun Li, Jingping Sun.

**Project administration**: Dan Wang, Jun Li, Jingping Sun.

**Resources**: Jun Li.

**Software**: Jun Li.

**Supervision**: Dan Wang, Jun Li, Jingping Sun.

**Validation:** Dan Wang.

**Visualization**: Dan Wang.

**Writing – original draft**: Dan Wang.

**Writing – review & editing**: Dan Wang, Jun Li, Jingping Sun.
